# YOUNG CAREGIVERS: ENGAGING FAMILIES AND BUILDING TRUST IN RESEARCH FOR CAREGIVERS OF ALL AGES

**DOI:** 10.1093/geroni/igae098.0965

**Published:** 2024-12-31

**Authors:** Andrea Kalvesmaki, Melinda Kavanaugh

**Affiliations:** VA Salt Lake City Health Care System, Salt Lake City, Utah, United States; University of Wisconsin Milwaukee, Milwaukee, Wisconsin, United States

## Abstract

The common definition of who is a “caregiver”, especially for older adults, is typically an adult family member, most likely a wife of an aging spouse or a daughter of an aging parent. However, estimates of 2022 Census data indicate that over 5 million young people under the age of 18 (young caregivers) may be caring for an aging adult, or an adult with a disability. The RAISE care act, while acknowledging children may be supporting care for other family members, does not outline strategies to work with these children or their families, or build the research infrastructure to identify unmet needs of families with young caregivers. Research on young caregivers in the US is a relatively new, emerging field. Here we present what is currently known about young caregivers in the US, including findings on the impacts of caregiving on youth outcomes, and important gaps in our knowledge base. Our work has identified key barriers to engaging these family members in research and family services, including state laws that prevent parents from disclosing the important roles these young people play in supporting aging family members. As researchers in the field, we also describe strategies we are using to connect with young caregivers to identify unmet needs, and build capacity to support these families, young caregivers, and their care recipients.

